# Investigating the Effects of Hydraulic Shear on *Scenedesmus quadricauda* Growth at the Cell Scale Using an Algal-Cell Dynamic Continuous Observation Platform

**DOI:** 10.3390/microorganisms13081776

**Published:** 2025-07-30

**Authors:** Yao Qu, Jiahuan Qian, Zhihua Lu, Ruihong Chen, Sheng Zhang, Jingyuan Cui, Chenyu Song, Haiping Zhang, Yafei Cui

**Affiliations:** 1 Department of Environmental Science, College of Environmental Science and Engineering, Tongji University, Shanghai 200092, China; 2111436@tongji.edu.cn (Y.Q.); qianjh90@126.com (J.Q.); chen_ruihong@ctg.com.cn (R.C.); shengzhang@tongji.edu.cn (S.Z.); cuijy@zjweu.edu.cn (J.C.); 1552271@tongji.edu.cn (C.S.); hpzhang@tongji.edu.cn (H.Z.); 2 Shanghai Waterway Engineering Design & Consulting Co., Ltd., Shanghai 200131, China; 3 Shanghai Academy of Environmental Sciences, Shanghai 200233, China; luzhihua.lu@foxmail.com; 4 Three Gorges Smart Water Technology Co., Ltd., Shanghai 200434, China; 5 Institute of Water Sciences, Zhejiang University of Water Resources and Electric Power, Hangzhou 310018, China; 6 Academy of Forensic Science, Shanghai 200063, China

**Keywords:** dynamic observation, *Scenedesmus quadricauda*, cellular growth, hydraulic shear, computer vision, backpropagation neural network

## Abstract

Hydraulic shear has been widely accepted as one of the essential factors modulating phytoplankton growth. Previous experimental studies of algal growth have been conducted at the macroscopic level, and direct observation at the cell scale has been lacking. In this study, an algal-cell dynamic continuous observation platform (ACDCOP) is proposed with a parallel-plate flow chamber (PPFC) to capture cellular growth images which are then used as input to a computer vision algorithm featuring a pre-trained backpropagation neural network to quantitatively evaluate the volumes and volumetric growth rates of individual cells. The platform was applied to investigate the growth of *Scenedesmus quadricauda* cells under different hydraulic shear stress conditions. The results indicated that the threshold shear stress for the development of *Scenedesmus quadricauda* cells was 270 µL min^−1^ (5.62 × 10^−5^ m^2^ s^−3^). Cellular growth was inhibited at very low and very high intensities of hydraulic shear. Among all the experimental groups, the longest growth period for a cell, from attachment to PPFC to cell division, was 5.7 days. Cells with larger initial volumes produced larger volumes at division. The proposed platform could provide a novel approach for algal research by enabling direct observation of algal growth at the cell scale, and could potentially be applied to investigate the impacts of various environmental stressors such as nutrient, temperature, and light on cellular growth in different algal species.

## 1. Introduction

Hydrodynamic force critically affects algal physiological and ecological characteristics such as cell division, cell volume, cell morphology, and photosynthetic characteristics [1,2]. These effects are particularly significant in aquatic ecosystems, where water movement can vary from gentle flows to turbulent currents. The effects of hydrodynamics on algae involve two key aspects: hydraulic flush, which removes algae from the system; and small-scale turbulence, which influences algal growth [3,4]. Small-scale turbulence can disrupt the microenvironment around algal cells, influencing nutrient uptake and metabolic processes.

Several studies investigating the effects of hydraulic shear on algal cells have been based on the nutrient flux hypothesis [5,6]. According to the hypothesis, the thickness of the “laminar boundary layer” on the surface of algal cells changes due to hydraulic shear, which alters the transport of nutrients and cellular metabolites through the cell wall, thereby affecting cellular growth [7]. For example, under high shear stress, the boundary layer becomes thinner, potentially enhancing nutrient diffusion but also increasing the risk of cell damage. Moreover, it has been found that small-scale turbulence plays an important role in the formation of *Microcystis* scum in aquatic ecosystems [8,9]. Li et al. suggested that turbulence was an essential factor driving aggregation of algal cells and morphological changes in algal colonies, and that colony size might be reduced under high turbulence intensities [10]. This phenomenon may be attributed to the mechanical disruption of colony integrity under turbulent conditions. Papoutsakis further noticed that small-scale eddies smaller than the cell size could be dissipated on the cell surface and transfer the energy to the particle, thereby causing more damage to cells [11]. This highlights the delicate balance between the beneficial and detrimental effects of hydrodynamics on algal cells.

Artificial Neural Networks (ANNs) have recently been applied to algal research, offering new tools for data analysis and prediction [12,13,14]. For instance, Manian et al. presented a semi-supervised method to label and classify hyperspectral images to differentiate blue-green algae and surface scum [15]. This approach leverages machine learning to improve the accuracy of algal identification in complex environmental samples. Similarly, Vinayagam et al. proposed an ANN model to predict adsorption of Cr (VI) by macroalgal spent biomass, demonstrating the potential of ANNs in environmental remediation studies [2]. Chen et al. advanced the field by establishing an improved sparrow search algorithm (FASSA-GRNN) to predict concentrations of brown tide algae cells [16]. Despite these advancements, challenges remain, particularly in capturing small-sized algal cells. This has led researchers to focus on algal colonies as the primary research material in many studies.

The increased use of electron microscopy has enabled in-depth investigations of cellular morphology and subcellular structures, marking a significant breakthrough in algal studies. For example, Fraisse et al. proposed that the response of phytoplankton to turbulence could be predicted from morphofunctional traits, suggesting a link between cell shape and hydrodynamic tolerance [17]. Medina et al. developed a visual detection system based on deep learning and computer vision algorithms to detect algae in underwater pipelines, showcasing the integration of technology into algal monitoring [5]. However, a lack of effective dynamic continuous observation methods for algal cells has resulted in an absence of cellular growth information, limiting further exploration of algal behavior under varying hydrodynamic conditions. Additionally, while research on colony types has provided valuable insights, little is known about the effects of small-scale turbulence on the growth mechanisms of algal species at the single-cell level.

To address these gaps, this study proposes a computer-vision-based algal-cell dynamic continuous observation platform (ACDCOP) designed to evaluate the effects of hydraulic shear on *Scenedesmus quadricauda* at the cell scale. The ACDCOP integrates high-resolution imaging with real-time data analysis, enabling precise monitoring of cellular responses to hydrodynamic forces. By applying this platform, the study aims to investigate the effects of hydraulic shear on algal growth at the single-cell level, providing new insights into the mechanistic underpinnings of algal behavior under dynamic water conditions.

## 2. Materials and Methods

### 2.1. Algae Cultivation

*Scenedesmus quadricauda* FACHB 44 was purchased from the Freshwater Algae Culture Collection of the Institute of Hydrobiology (FACHB), Chinese Academy of Sciences, Wuhan, China. Algae cells were cultivated in growth BG-11 medium (Table S1) and grown at 25 ± 2 °C [18] under a 12L:12D regime (incident light intensity: 30 µmol m^−2^ s^−1^) in a light incubator (HGZ-250, Huitai Co., Shanghai, China). During the cultivation period, all the flasks were shaken by hand two times every day to avoid agglomeration.

### 2.2. Algal-Cell Dynamic Continuous Observation Platform

#### 2.2.1. Equipment Configuration of ACDCOP

**Figure 1:**
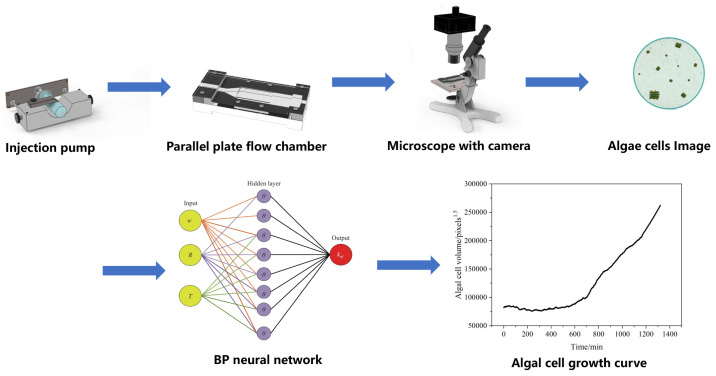
Framework and workflow of ACDCOP.

Figure 1 shows the framework of ACDCOP, which consists of a parallel-plate flow chamber (PPFC, self-designed) to immobilize the algal cell, an injection pump (Lange injection pump controller model TJ-3A, actuator W0109-1B, Baoding, China) to control nutrient conditions and hydraulic shear stress in PPFC, an optical microscope (Olympus CKX41, Tokyo, Japan), a camera (SONY NEX-VG20EH, Tokyo, Japan), a computer (Processor Intel i7-2600, Santa Clara, CA, USA), a display card (Nvidia GeForce 405, Santa Clara, CA, USA), and a video capture card (HD100C Full HD, Tokyo, Japan). Fibronectin from human plasma (4 µg mL^−1^, Aladdin, Shanghai, China) is placed on the inner surface of PPFC, where *Scenedesmus quadricauda* cells are attached and grow under controlled conditions. Cell images captured by the camera are used as input to a computer vision algorithm featuring a pre-trained backpropagation (BP) neural network to quantitatively estimate the volume of algal cells in each image.

Throughout the experimental period, cell images were captured at 5-minute intervals by the camera. A microscope calibration scale (0.01 mm DIV, Aosvi, Suzhou, China) was used to determine the conversion ratio between pixels of algal cells in the image and sizes of real algal cells (μm). In the study, the ratio was 0.30 ± 0.001 μm/pixel.

#### 2.2.2. Image Processing and Computer Vision Algorithm

Bitmap images of algal cells with 8-bit color depth were used as the training data. Manual classification was performed using the original images (Figure S1). The image data and output values were input into the training function of a BP neural network. The function was used for cell image identification based on the MATLAB 2012 toolbox’s BP neural network. The original data were randomly divided into three groups, namely, training samples, test samples, and calibration samples, in proportions of 70%, 15%, and 15%. The input layer was a single linear node and the output layer was a cell/background decision matrix. The Levenberg–Marquardt method was used for optimization training of the algorithm, wherein the algorithm convergence evaluation condition was set to a mean square error of 10^−6^. The RGB data of the cell images from the camera was read into the trained 3-layer BP neural network. Each pixel was determined by the neural network and outputted as a black-and-white image. The positions and sizes of algal cells in the image were then identified.

### 2.3. Experimental Conditions

Flow turbulence intensity is characterized by many parameters, such as energy dissipation rate ε [19,20], Reynolds number Re [21], and shear stress τ [11]. The turbulent energy dissipation rate is a critical variable in studying the effects of small-scale fluid flow on microbial physiology at the cellular level [22]. It reflects energy transfer in a turbulent environment from large scale to small scale, and its eventual dissipation due to molecular adhesion.

In the study, the flow gradients were set to 30, 60, 90, 120, 150, 180, 210, 240, 270, 300, 360, and 420 uL min^−1^. The turbulence energy dissipation rate ε was simulated with the CFD (Computational Fluid Dynamics) software FLUENT ansys 14.0 (Ansys Fluent Inc., Lebanon, NH, USA). Values of ε for oceans, rivers, and lakes in natural ecosystems range from 2.8 × 10^−11^ to 4.7 × 10^−2^ m^2^ s^−3^, 10^−6^ to 10^−3^ m^2^ s^−3^, and 10^−9^ to 10^−6^ m^2^ s^−3^, respectively [23,24]. As shown in Table S2, the range of ε for the present study was set to 6.2 × 10^−6^–7.5 × 10^−5^ m^2^ s^−3^, generally covering the natural conditions of rivers and lakes.

The cultivation period was set to 7 days for each experiment under per flow condition after many test experiments. Each experiment was conducted in triplicate. A specific microscopic field of view was selected, containing 5 to 10 algal cells to balance the number of sample cells and the clarity of cell image. Although in each experiment 15 to 30 algal cells were observed in total, some of the cells were not stably attached to PPFC and swung with the flow, affecting the image quality; some cells were even flushed away by flow during the observation. Moreover, some of the fixed cells began to divide shortly after the experiment started and no obvious growth process was observed. Therefore, only the three algae cells with the longest cultivation periods in each experiment were selected for presentation in the paper.

## 3. Results

### 3.1. The Volume Growth Model of Individual Cells

The fitting of the volume growth curve for algal cells involved slight fluctuation due to image noise. The exponential smoothing method was used for the growth curves, as follows:

(1) $$S_{t}^{1}=\alpha Y_{t}+\left(1-\alpha \right)S_{t-1}^{1}$$

where $S_{t}^{1}$ is one exponential smoothing value at time *t*; $Y_{t}$ is the time series; and α is the weight coefficient, with 0<α<1. α reflects the ability of the model to correct the error. The magnitude of the smoothing increases as the value of α increases. The value of α was chosen according to the changing characteristics of the time series. In the study, the fluctuation in the volume growth curves for *Scenedesmus quadricauda* cells was not significant. Therefore, the α value was set to 0.1. The algal volume was represented by a third-degree polynomial fit to the growth curve under different shear stress conditions, expressed as follows:

(2) $$V=at^{3}+bt^{2}+ct+d$$

where V is the volume of algal cells (μm^3^); t is time (min); and a, b, c, and d are regulation coefficients.

The primary factors influencing cell growth are the initial condition of the cell and the effects of internal and external factors on the cell [25]. The coefficient *d* reflects the initial cell volume of an algal cell. The effects of environmental factors on cellular biomass are generally linear [26]; therefore, *ct* indicates the influence of external factors on algal growth. $at^{3}+bt^{2}$ reflects the influence of internal factors on algal cells, which mainly indicate biochemical responses such as enzymatic reactions [27]. Cells are also more strongly influenced by enzymatic reactions than by the external environment; therefore, the influence of internal factors on cell growth is a higher-order variable of t [28]. The parameter values for the fitted growth curves for algal cells under different shear stress conditions are provided in Table 1.

### 3.2. Effect of Hydraulic Shear on Scenedesmus quadricauda

#### 3.2.1. Effect on Cell Growth

**Figure 2:**
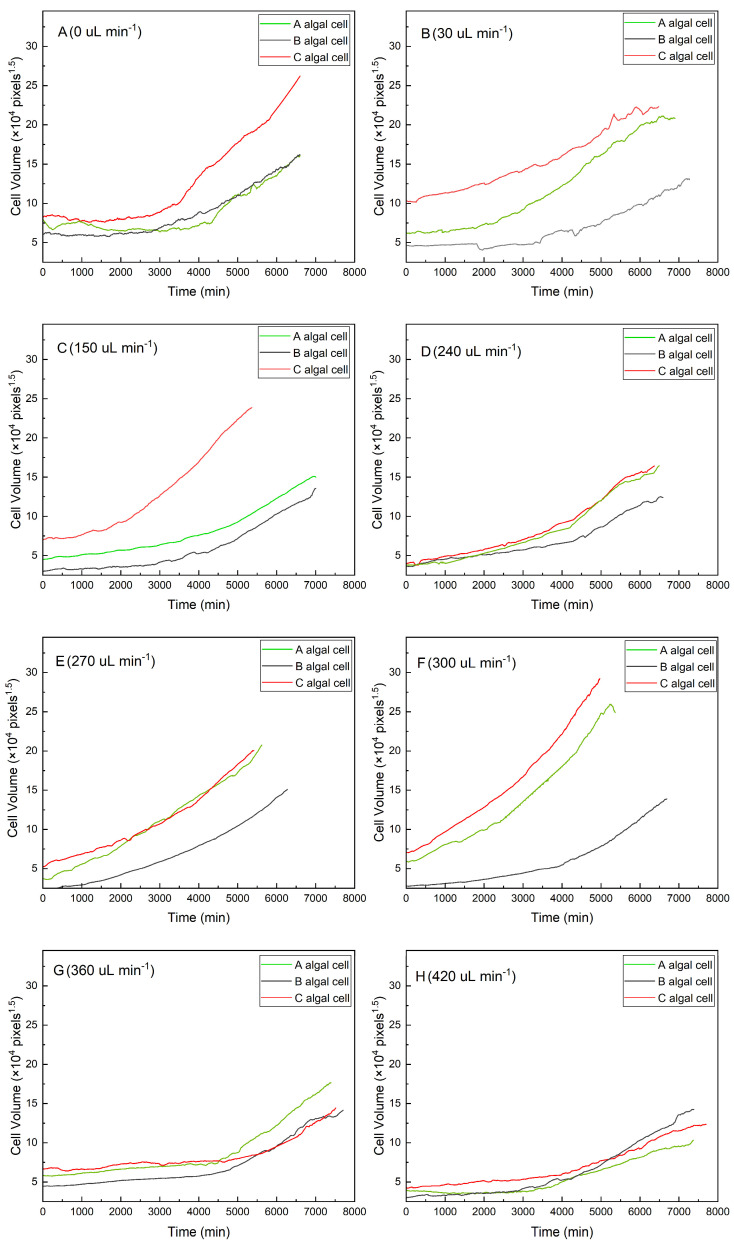
Growth curves for individual algal cells under different flow rates: (**A**) 0 μL min^−1^, (**B**) 30 μL min^−1^, (**C**) 150 μL min^−1^, (**D**) 240 μL min^−1^, (**E**) 270 μL min^−1^, (**F**) 300 μL min^−1^, (**G**) 360 μL min^−1^, and (**H**) 420 μL min^−1^.

As shown in Figure 2, algal cells with a larger initial volume had a shorter lag growth phase under the same flow conditions (red curves in Figure 2A–C,F,H). In contrast, algal cells with a smaller initial volume required a longer adaptation period, during which the growth curve was almost horizontal and lasted longer (gray curves in Figure 2A–C,E,F). The results suggested that algal cells with a large initial volume could adapt quickly to a new environment. The smaller cells, however, showed a much higher growth rate after a longer lag phase (gray curves in Figure 2A,B,F–H).

**Figure 3:**
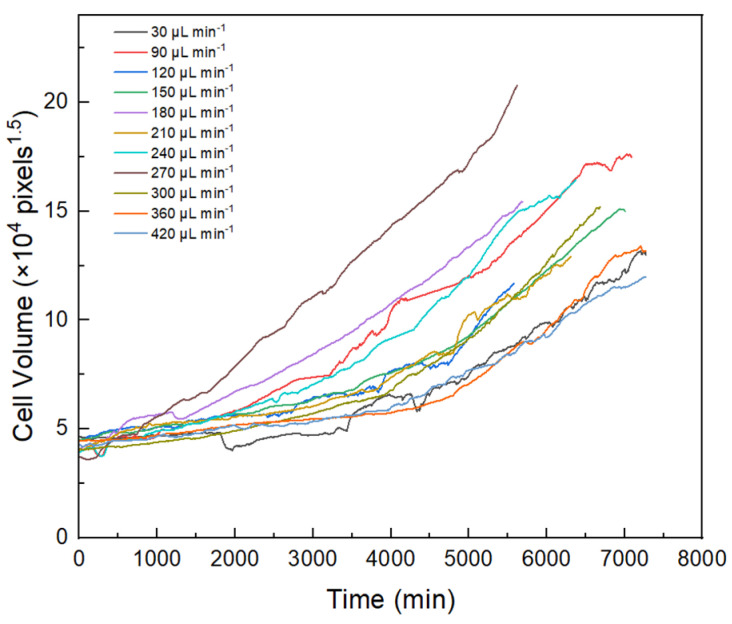
Growth curves for algal cells of similar initial volume (27 µm^3^) under different flow rates.

To compare changes in algal growth rates under different shear stress conditions, algal cells with a similar initial volume (27 μm^3^) were selected for analysis in the study (Figure 3). The growth rates of algal cells generally increased with increased shear stress. At flow rates of 30, 360, and 420 µL min^−1^, the algal cells showed slow or no growth, implying that the algal cells might be less able to adapt to the environment at very low and very high intensities of hydraulic shear. In contrast, when the flow rate was 270 µL min^−1^, the algal cells had a shorter adaptation time after their attachment to PPFC. Moreover, the algal cells rapidly adapted to the new environment at this flow rate, and the growth rate was significantly higher than that of the algal cells at other flow rates. The results suggested that the flow rate of 270 µL min^−1^ (5.62 × 10^−5^ m^2^ s^−3^) might be the threshold shear stress for the growth of *Scenedesmus quadricauda* under hydraulic shear.

#### 3.2.2. Effect on Cell Volume at Division

The end of each growth curve shown in Figure 2 indicates the moment when algal cell division began. Volumes at division with different initial volumes showed some variation under the same shear stress conditions. As an example, when the flow rate was controlled at 150 µL min^−1^, as shown in Figure 2C, the volumes at division of the three cells were 91.61, 101.89, and 160.57 μm^3^, respectively, and their initial volumes were 19.99, 30.25, and 47.28 μm^3^, respectively, indicating that the volume at division was larger if the initial volume was larger. The volumes at division of single cells under different shear stress conditions exhibited considerable variation. It was found that cells with similar initial volumes did not have similar volumes at division under different shear stress conditions, as shown in Figure 3. Volume at division reached a maximum of 140.07 μm^3^ at a flow rate of 270 µL min^−1^, indicating that a shear stress of 270 µL min^−1^ (5.62 × 10^−5^ m^2^ s^−3^) was most favorable to the growth of *Scenedesmus quadricauda* cells.

**Figure 4:**
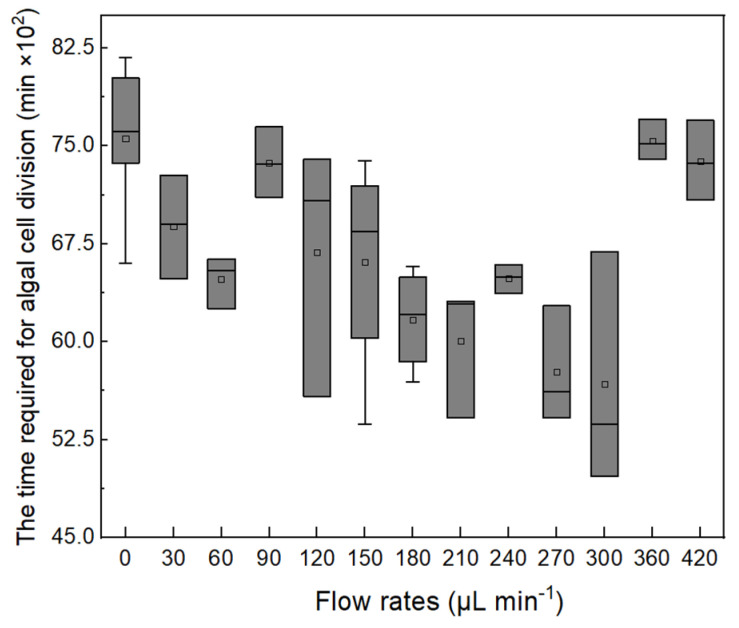
Box plot of cell growth periods under different flow rates.

In the study, the ACDCOP recorded the time from when the cell attached to PPFC to the beginning of its division. Cell growth periods under different shear stress conditions exhibited considerable variation, as shown in Figure 4. The shortest growth period was found at a flow rate of 180–300 µL min^−1^, indicating that suitable hydraulic shear could accelerate cell growth. Moreover, the longest growth period was found to be 8175 min (5.7 d) in the study.

## 4. Discussion

The ACDCOP proposed in this study provides a novel approach for algal research by enabling direct observation of algal growth at the cell scale. Volume growth curves for single cells can be established to investigate the effect of hydraulic shear on cell growth. Previous experimental studies on algal growth have been conducted at the macroscopic level; these indicated that algal growth is modulated by hydraulic shear [3,7,29]. A threshold level of shear stress was found to exist, below which algal growth is promoted and over which it is inhibited [3]. Results obtained in the present study were consistent with these findings, and the threshold shear stress was found to be 270 µL min^−1^ (5.62 × 10^−5^ m^2^ s^−3^) under the experimental conditions described in this paper. Our results also supported the “laminar boundary layer” hypothesis which assumes that transport of nutrient and cellular metabolites through the cell wall is facilitated due to reduced diffusive layer thickness under moderate shear stress [7,30], and the hypothesis which assumes that higher levels of shear stress have a negative impact on cell physiology and morphology [31], because hydraulic shear can be considered the only cause of the differences in cellular growth among the experimental groups in the present study.

The phenomenon of fluid-mechanical damage to algal cells under high hydraulic shear stress has been reported in the literature [11,32]; however, this was not observed in this study because the algal cells attached to the surface of PPFC could no longer be immobilized stably or even be flushed away under high flow rates. This represents the limitation of ACDCOP. Further studies should therefore be conducted to enhance the bond strength of the fibronectin used for PPFC.

ACDCOP could potentially be applied to investigate the impact of nutrient (by controlling nutrient concentrations through the injection pump), temperature, and light on cell growth in other algal taxa such as cyanobacteria and diatoms, as long as their morphological images can be captured by the camera in ACDCOP. However, it should be noted that the computer vision algorithm integrated into the current ACDCOP framework was trained and calibrated using an imaging system with 13.6-megapixel resolution (video capture at 1080p) and the model organism *Scenedesmus quadricauda*. Consequently, the algorithm will require retraining if a new imaging system is incorporated or new algal species used for investigation.

## 5. Conclusions

An algal-cell dynamic continuous observation platform (ACDCOP) is proposed in the study, consisting mainly of a parallel-plate flow chamber (PPFC), an injection pump, an optical microscope, a computer, a display card, and a video capture card. Cell images captured by the camera are used as input to a computer vision algorithm featuring a pre-trained BP neural network, to quantitatively evaluate the volumes and volumetric growth rates of individual cells. The platform was applied to investigate the growth of *Scenedesmus quadricauda* cells under different hydraulic shear stress conditions. The results indicated that the threshold shear stress for the development of *Scenedesmus quadricauda* cells was 270 µL min^−1^ (5.62 × 10^−5^ m^2^ s^−3^). Cellular growth was inhibited at very low and very high intensities of hydraulic shear, consistent with findings reported in the literature. Among all the experimental groups, the longest growth period for a cell, from attachment to PPFC to cell division, was found to be 5.7 days. Cells with larger initial volumes produced larger volumes at division. The platform could provide a novel approach for algal research by enabling direct observation of algal growth at the cell scale, and could potentially be applied to investigate the impact of various environmental stressors such as nutrient, temperature, and light on cell growth in different algal species.

## Figures and Tables

**Table 1:** The parameter values of fitted growth curves for algal cells under different flow rates.

Flow Rates (µL Min^−1^)	*a* (×10^−10^)	*b* (×10^−3^)	*c* (×10^−2^)	*d*	*R* ^2^
0	−0.10 ± 0.32	3.7 ± 3.9	−1.0 ± 0.80	53 ± 10	0.989 ± 0.012
30	−2.0 ± 3.2	3.9 ± 3.6	0.20 ± 0.20	50 ± 19	0.993 ± 0.003
60	0.80 ± 2.1	1.7 ± 1.6	0.00 ± 0.60	37 ± 6	0.996 ± 0.001
90	1.0 ± 1.7	0.90 ± 1.4	0.20 ± 0.20	28 ± 8	0.997 ± 0.001
120	3.4 ± 1.8	−1.5 ± 0.22	0.40 ± 0.40	24 ± 6	0.987± 0.005
150	2.0 ± 0.60	0.22 ± 0.80	0.00 ± 0.40	27 ± 6	0.997 ± 0.002
180	0.40 ± 3.6	1.3 ± 1.8	0.40 ±0.40	32 ± 7	0.998 ± 0.000
210	0.80 ± 1.4	0.68 ± 1.4	0.20 ± 0.20	21 ± 2	0.995 ± 0.005
240	1.6 ± 2.2	−1.1± 2.0	0.40 ± 0.40	26± 2	0.993 ± 0.001
270	1.6 ± 1.4	1.0 ± 1.0	0.80 ± 0.40	25± 11	0.999 ± 0.001
300	4.2 ± 1.8	−0.10 ± 1.5	1.2 ± 0.80	34 ± 14	0.999 ± 0.001
360	3.2 ± 1.4	−2.0 ± 1.4	0.60 ± 0.40	37 ± 5	0.975 ± 0.026
420	−0.20 ± 1.3	1.7 ± 1.3	−0.40 ± 0.40	28 ±4	0.994 ± 0.003

## Data Availability

The original contributions presented in this study are included in the article. Further inquiries can be directed to the corresponding author.

## Supplementary Materials

The following supporting information can be downloaded at: https://www.mdpi.com/article/10.3390/microorganisms13081776/s1, Figure S1. Training sample of Background-Algal cells-RGB. Figure S2. Volume change of *Scenedesmus quadricauda* cells observed during cultivation (1, 2, 3, 4, 5, and 6 represent the images of the algae cells at each 6-hour interval). Figure S3. Cell image processing (Left: raw image; Right: cell size recognized by the computer vision algorithm, 40× objective, 1920 × 1080 resolution). Table S1. The medium formula of BG11. Table S2. Shear stress at different flow rates.
