# Supplementary material for: Investigating the Effects of Hydraulic Shear on Scenedesmus quadricauda Growth at the Cell Scale Using an Algal-Cell Dynamic Continuous Observation Platform
Source: Microorganisms. 2025 Jul 30;13(8):1776. doi: 10.3390/microorganisms13081776 (PMC12388814; doi:10.3390/microorganisms13081776)
Supplement: Supplementary file 1 [file microorganisms-13-01776-s001.zip › microorganisms-3738897-supplementary.pdf]

microorganisms

MDPI

Supplementary Materials: Supplementary Data

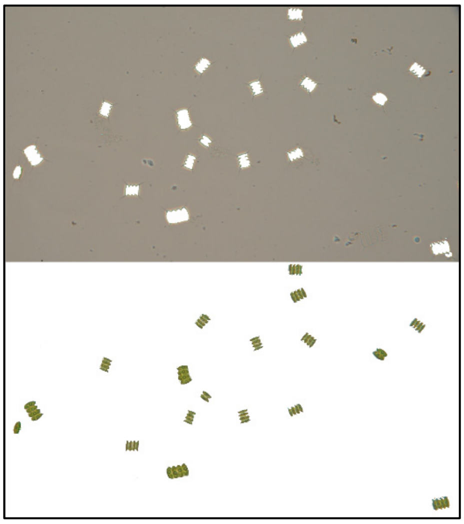

Figure S1. Training sample of Background-Algal cells-RGB.

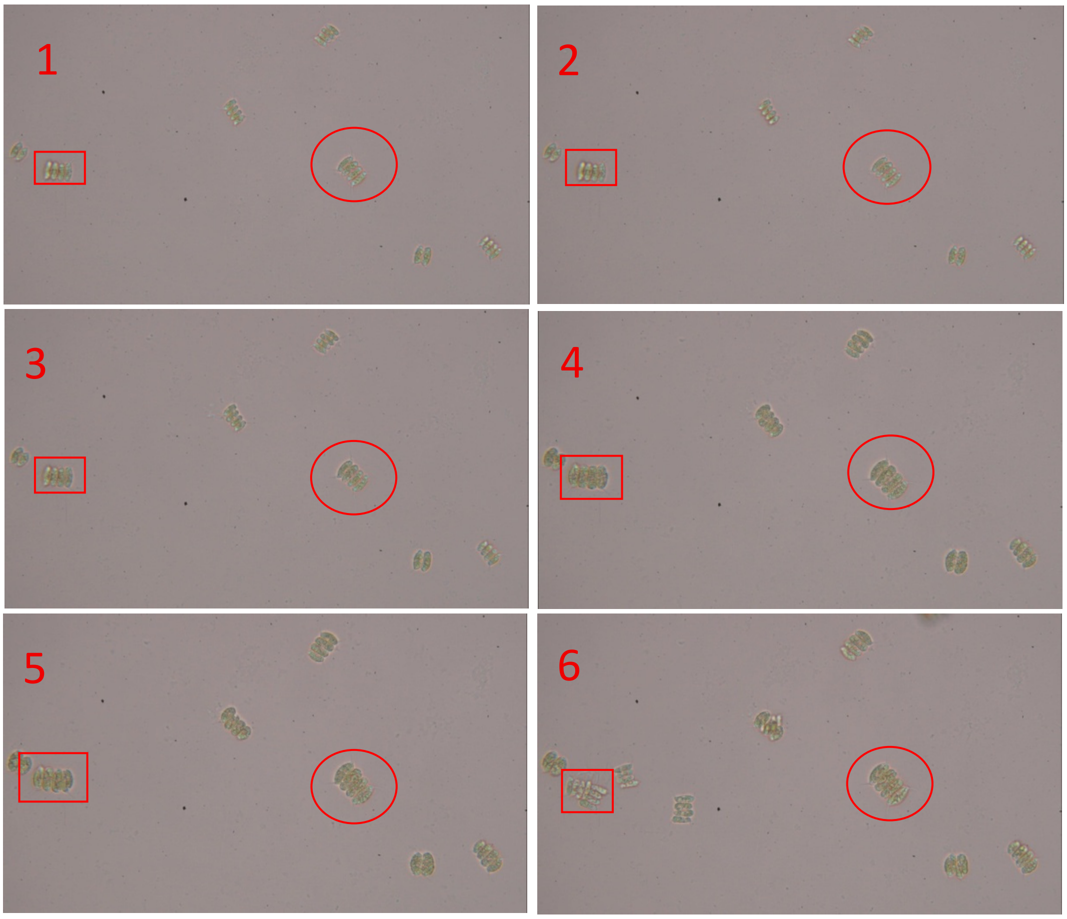

Figure S2. Volume change of Scenedesmus *quadricauda* cells observed during cultivation (1, 2, 3, 4, 5, and 6 represent the images of the algae cells at each 6-hour interval).

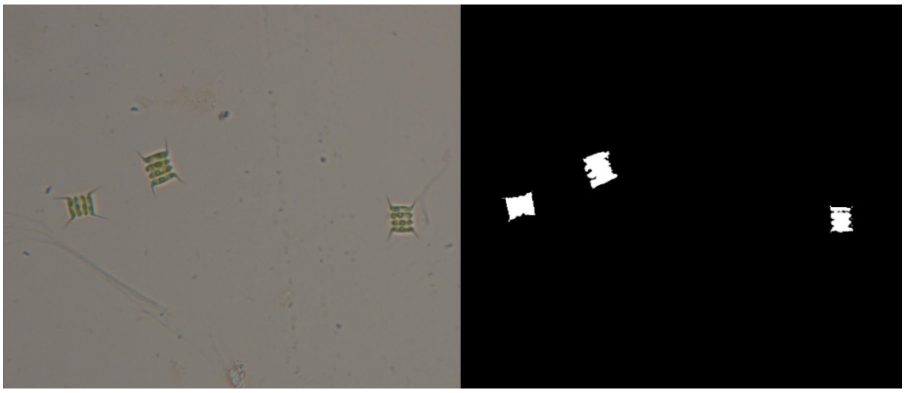

Figure S3. Cell image processing (Left: raw image; Right: cell size recognized by the computer vision algorithm, 40x objective, 1920 × 1080 resolution).

Table S1. The medium formula of BG11.

No.	Component	Content
1	$NaNO_3$	1.5 g L^{-1}
2	K_2HPO_4	0.04 g L^{-1}
3	$MgSO_4·7H_2O$	0.075 g L^{-1}
4	$CaCl_2·2H_2O$	0.036 g L^{-1}
5	Citric acid	0.006 g L^{-1}
6	Ferric ammonium citrate	0.006 g L^{-1}
7	$EDTANa_2$	0.001 g L^{-1}
8	Na_2CO_3	g L^{-1}

Table S2. Shear stress at different flow rates.

**Flow rate (µl min^{-1})**	0	30	60	90	120	150	180	210	240	270	300	360	420
**Shear stress (Pa)**	0	5.6	11.2	16.8	22.4	28	33.6	39.2	44.8	50.4	56	67.2	78.4
**Turbulence energy dissipation rate $\times 10^{-6}$ m^2 s^{-3}**	0	6.2	12.5	18.8	25.0	31.2	37.5	43.8	50.0	56.2	62.5	68.8	75.0

**Flow rate (µl min^{-1})**	30	60	90	120	150	180	210	240	270	300	360	420
**Shear stress (Pa)**	5.6	11.2	16.8	22.4	28	33.6	39.2	44.8	50.4	56	67.2	78.4
**Turbulence energy dissipation rate $\times 10^{-6}$ m^2 s^{-3}**	6.2	12.5	18.8	25.0	31.2	37.5	43.8	50.0	56.2	62.5	68.8	75.0
